# Predictors of Fighting Ability Inferences Based on Faces

**DOI:** 10.3389/fpsyg.2018.02740

**Published:** 2019-01-15

**Authors:** Vít Třebický, Jitka Fialová, David Stella, Klára Coufalová, Radim Pavelka, Karel Kleisner, Radim Kuba, Zuzana Štěrbová, Jan Havlíček

**Affiliations:** ^1^Faculty of Science, Charles University, Prague, Czechia; ^2^Applied Neurosciences and Brain Imaging, National Institute of Mental Health, Klecany, Czechia; ^3^Faculty of Physical Education and Sport, Charles University, Prague, Czechia

**Keywords:** perception, formidability, aggressiveness, strength, anaerobic performance, vital capacity, body composition, beardedness

## Abstract

Facial perception plays a key role in various social interactions, including formidability assessments. People make relatively accurate inferences about men's physical strength, aggressiveness, and success in physical confrontations based on facial cues. The physical factors related to the perception of fighting ability and their relative contribution have not been investigated yet, since most existing studies employed only a limited number of threat potential measures or proxies. In the present study, we collected data from Czech Mixed Martial Arts (MMA) fighters regarding their fighting success and physical performance in order to test physical predictors of perceived fighting ability made on the basis of high-fidelity facial photographs. We have also explored the relationship between perceived and actual fighting ability. We created standardized 360° photographs of 44 MMA fighters which were assessed on their perceived fighting ability by 94 raters (46 males). Further, we obtained data regarding their physical characteristics (e.g., age, height, body composition) and performance (MMA score, isometric strength, anaerobic performance, lung capacity). In contrast to previous studies, we did not find any significant links between the actual and the perceived fighting ability. The results of a multiple regression analysis have, however, shown that heavier fighters and those with higher anaerobic performance were judged as more successful. Our results suggest that certain physical performance-related characteristics are mirrored in individuals' faces but assessments of fighting success based on facial cues are not congruent with actual fighting performance.

## Introduction

Male intra-sexual competition is considered an important factor of selective pressure (Puts, 2010; Třebický et al., 2012; Hill et al., 2013; Sell et al., 2017), because it is associated with access to resources via rise in social hierarchy and consequently also with broader mating opportunities. Evidence from various cultures (e.g., von Rueden et al., 2008) and ancestral societies (Walker, 2001) suggests that incidence of physical confrontations in humans is comparable to non-human species (Ellis, 1995). Benefits that can be gained in such confrontations must be, however, always weighed against potential costs, which may include injuries or even death. Decision whether to flee or fight is therefore frequently taken before an actual physical confrontation takes place, which means that one of the opponents often surrenders without a fight (Sell et al., 2012). Individuals who are good at assessing their chances to win are likely to gain a selective advantage. We may thus expect that perceptual and/or cognitive adaptations for the assessments of one's own and others' fighting ability have evolved.

Recent research shows that humans are capable of inferring fighting ability from facial, body, and vocal cues (Sell et al., 2009, 2010; Puts et al., 2012; Třebický et al., 2013; Little et al., 2015; Raine et al., 2018). Current studies tend to focus on investigating the relationship between the individual components of threat potential, such as body size, upper-body strength, or fighting success, and facial perception (Sell, 2016). One cross-cultural study demonstrated that people can assess upper-body strength and fighting ability of males from facial photographs alone (Sell et al., 2009). Several other studies have investigated the association between hand-grip strength—a frequently used proxy for upper-body strength (Wind et al., 2010)—and various characteristics perceived from faces. It has been repeatedly shown that physically stronger men receive higher ratings of dominance, masculinity, and attractiveness (Fink et al., 2007; Windhager et al., 2011; Geniole and McCormick, 2015; Gallup and Fink, 2018). When 3D facial stimuli were used, Holzleitner and Perrett (2016) found an association between actual and perceived strength, but weaker than in earlier investigations. Results of this study also suggest that perceived strength was independently predicted by the amount of muscle and fat, which mediated the effect of actual strength on the perceived strength (Holzleitner and Perrett, 2016). A recent study revealed that men's perceived “facial threat potential”—derived from dominance, strength, and weight ratings—is related to scores of “actual threat potential,” as based on a composite measure of hand-grip strength, weight, and height (Han et al., 2017).

Another line of research investigates the association between actual fighting ability and facial perception by employing Mixed Martial Arts (MMA) fighters and their fighting success score. When fight outcomes were assessed from faces of particular pairs of MMA fighters with known fight outcome, the actual winners were selected as more likely to win a fight, as being more aggressive, stronger, and more masculine than the losers (Little et al., 2015). A rating study by Třebický et al. (2013) showed that perceived aggressiveness of MMA fighters is associated with their fighting success. Moreover, actual fighting ability was also linked to perceived fighting ability, but only in heavyweight fighters. However, the factors responsible for the perception of fighting ability, including their relative contribution, has not been investigated yet. This is partly due to the fact that most existing studies employ only a limited number of threat potential measures and/or proxies.

To explore these issues, we collected detailed data on Czech MMA fighters, regarding their actual fighting ability, and those physical performance measurements which were considered important in previous studies focused on the performance of MMA fighters (e.g., Lenetsky and Harris, 2012; Alm and Yu, 2013). We have chosen MMA as an analog to real-life physical confrontations because it combines various fighting styles used in other combat sports and blends them into a unique multielement martial art. It employs a wide variety of techniques: opponents fight in a standing position, where they rely on punches and kicks (much like in boxing, kick-boxing, and Muay Thai), but also on the ground, where they wrestle and grapple (using techniques from e.g., Brazilian Jiu-Jitsu, Judo, Greco-Roman wrestling, and freestyle wrestling). The extremely dynamic nature of MMA fights involves both repeated explosive movements and submaximal dynamic work, that is, a combination of high anaerobic and aerobic demands (Lenetsky and Harris, 2012). For these reasons, body composition (Boileau and Lohman, 1977; Braswell et al., 2010), aerobic endurance (Yoon, 2002; Radovanovic et al., 2011; Durmic et al., 2017), maximum strength, and anaerobic capacity (AC) (La Bountry et al., 2011) all play an important role in maintaining performance throughout the fight.

To cover a broad range of physical factors which might affect perceived fighting ability, we collected data on overall body strength (measured as the maximal isometric strength of hands, arms, legs, trunk, and neck), endurance (using lung capacity measurements), AC (using the Wingate test), and body composition (data on body weight, body fat mass, muscle mass, and bone mass).

Further, it has been shown that men's beardedness, while having no effect on fighting outcomes in competitions (Dixson et al., 2018), is linked to judgements of higher levels of masculinity (Dixson et al., 2017), dominance (Muscarella and Cunningham, 1996; Neave and Shields, 2008; Dixson and Vasey, 2012; Saxton et al., 2016; Sherlock et al., 2017), and aggressiveness (Muscarella and Cunningham, 1996; Neave and Shields, 2008; Dixson and Vasey, 2012; Geniole and McCormick, 2015). For this reason, we have also explored the effect of facial hair on the perception of fighting ability.

Most existing studies tended to rely on static frontal facial photographs of varying quality and standardization, which convey a limited amount of visual information regarding overall facial morphology (Danel et al., 2018). To overcome these issues, we collected highly standardized 360° view photographs of heads, which provide more visual information. These were then used to investigate the relationship between the perception of fighting ability and various measures of athletes' physical performance.

## Materials and Methods

All procedures followed were in accordance with ethical standards of the relevant committee on human experimentation and with the Helsinki Declaration. The study was approved by the Institutional Review Board of the National Institute of Mental Health, Czech Republic (Ref. num. 28/15). All participants were informed about the goals of the study and approved their participation by signing informed consent.

### Participants

#### Targets

In total, we obtained photographs and data on physical performance from 44 MMA athletes (mean age = 26.7, *SD* = 5.91, range = 18–38); all residents of the Czech Republic. They were recruited via social media advertisements, leaflets distributed at local MMA tournaments, at gyms, and with the help of Mixed Martial Arts Association Czech Republic (MMAA). They were reimbursed for their participation with 400 CZK (~15 EUR). To obtain information about their fighting success rate, we computed their actual fighting ability as the proportion of wins relative to the total number of fights. Hereafter, the term “actual fighting ability” refers to their actual success in competition.

#### Raters

Photographs were judged by 46 male (mean age = 21.96 years, *SD* = 2.56, range = 19–29) and 48 female raters (mean age = 22.29 years, *SD* = 3.56, range = 18–38), mainly Charles University students, who were recruited via social media advertisements and mailing list of participants established in previous studies. The participants received 100 CZK (~4 EUR) as a compensation for their participation and a debriefing leaflet which explained the purpose of the study.

### Stimuli Collection

#### Photographs Acquisition and Setting

Photographs were captured with 24 megapixels full-frame (35.9 × 24 mm CMOS sensor, a 35 mm film equivalent) digital SLR camera Nikon D610 equipped with a fixed focal length lens Nikon AF-S NIKKOR 85 mm f/1.8 G (Třebický et al., 2016). Exposure values were set to ISO 100, shutter speed 1/200 s and aperture f11 in all photographs. Photographs were shot into 14-bit uncompressed raw files (NEF) and AdobeRGB color space. Color calibration was performed by using X-Rite Color Checker Passport color targets and a white balance patch photographed at the beginning of each session. The camera was mounted in portrait orientation directly on the light stand which also carried a strobe light positioned 125 cm from the participant. The aim of this setup was to achieve a perception close to the social interpersonal distance (Hall, 1966; Baldassare and Feller, 1975; Sorokowska et al., 2017), to maintain a constant distortion of perspective (Třebický et al., 2016; Erkelens, 2018), and to avoid potential perception bias based on interpersonal distance (Bryan et al., 2012). Camera's distance from each participant was checked with a digital laser rangefinder (Bosch PLR 15). Camera's height was adjusted individually for each participant (target) so as to center his head in the middle of the frame, and the focus point was set on participant's eye. This setting of camera's distance, focal length, and sensor size yielded a 35 × 53 cm field of view (23.85° angle of view).

**Figure 1:**
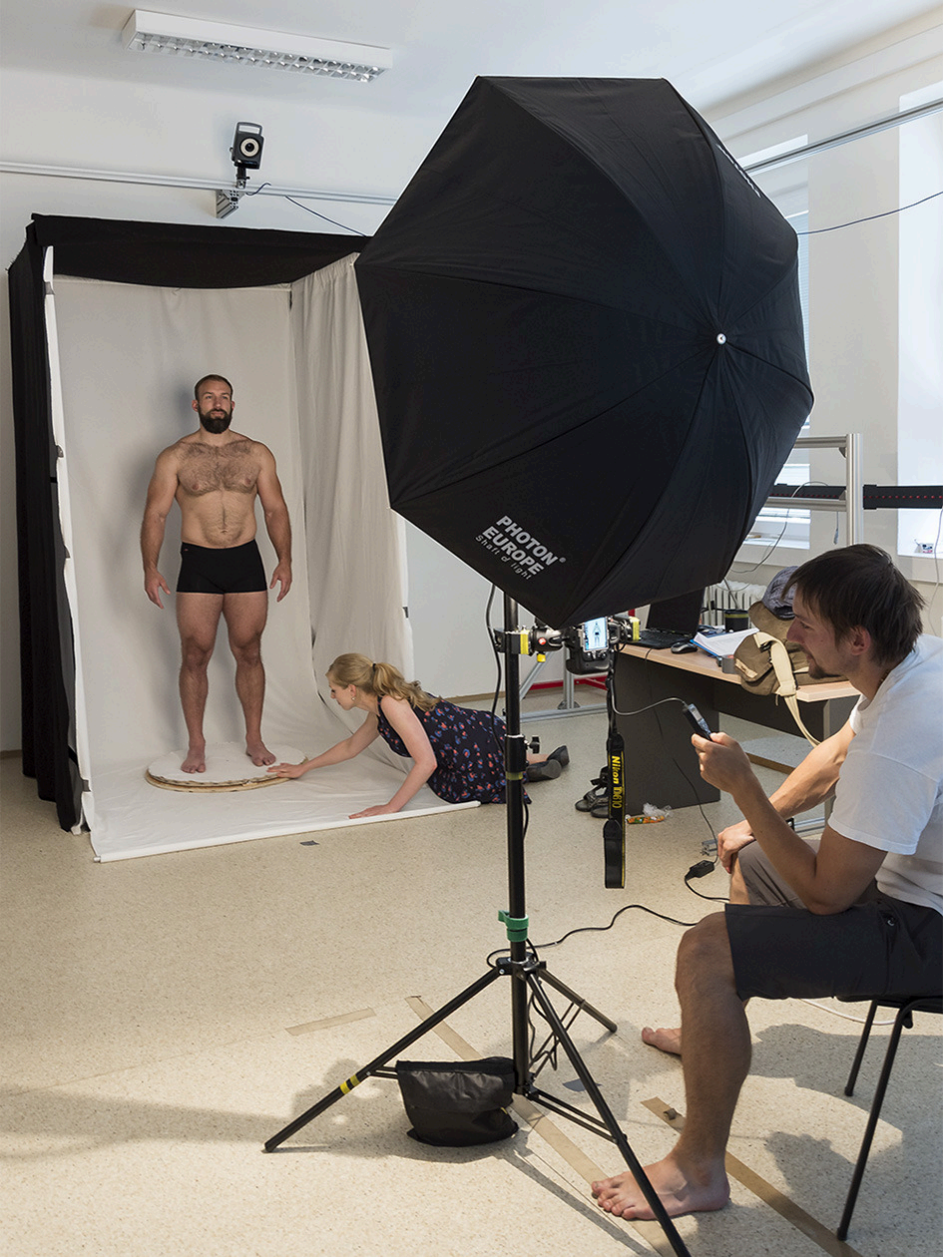
Illustrative image of photograph acquisition setup. Photograph by Jitka Fialová, published with informed and written consent of depicted participant and co-authors.

Participants were seated on a bar stool and asked to sit straight with hands hanging freely alongside their body. They were photographed in black underwear shorts we provided them with (i.e., they wore no T-shirts) and without any adornments, such as glasses or jewelry. They were instructed to look directly into the camera, adopt a neutral expression, and retain this position through whole photographing session. To capture 360° images of targets' head, a stool was placed on a turning platform which could be manually rotated by 10° in 36 steps, see Figure 1 for illustration. This resulted in 36 photographs for each participant. The platform was placed in a purpose-built portable photographic booth to control for any changes in ambient light and for color reflections (Rowland and Burriss, 2017; Thorstenson, 2018). We took two full rotations of each participant to obtain one full set of high-quality photographs (to eliminate possible movements between shots, blinks etc.).

Standardized lighting conditions and uniform exposure across the whole scene were ensured by using one 800 W studio strobe (Photon Europe MSN-800) with a white reflective umbrella used as a light diffuser (Photon Europe, 109 cm diameter) mounted onto a 175 cm high light stand, tilted 10° downwards toward the booth. Correct lighting exposure was checked before each session with a digital light meter (Sekonic L-308S). For further details on the photograph acquisition procedure, see Třebický et al. (2018).

#### Stimuli Processing and Building 360° Head Rotations

Final sets of 36 photographs of full 360° head rotation for each participant were selected and postprocessed in Adobe Lightroom Classic CC (Version 2017, Adobe Systems Inc.). First, we converted photographs into DNG raw files, then we built DNG color calibration profiles and applied them to all photographs. Exposure across all selected photographs was verified in three background areas around the head (above, left, right) and eventual slight differences in exposure were manually adjusted to the same level. Subsequently, the calibrated photographs were exported into lossless 16-bit AdobeRGB TIFF files in real size of 35 × 53 cm and 168 PPI. This allowed us to present images of participants' heads in their real-life size.

Photographs were aligned so that each participant's head was positioned in the center of each frame with eyes on the same horizontal line in all pictures. Final photographs were batch-cropped to 2,095 × 2,305 side ratio to fit head rotations of all participants. All photographs were subsequently converted into sRGB color space and exported as 8-bit JPEG files (2,095 × 2,305 px @ 168 ppi).

We built the 360° head rotations with Sirv (www.sirv.com, Magic Toolbox Limited), an online suite for creating and managing image spins. Photographs of all target participants were uploaded, and individual spins created.

#### Rating Sessions

Rating sessions took place in a quiet perception lab under standardized conditions. Raters were seated 125 cm from the screen, i.e., at the distance at which the photographs were captured, so as to approximate a social interpersonal distance (Sorokowska et al., 2017) and thereby increase the ecological validity of the rating session.

Ratings were carried out on a 27″ Dell U2718Q UltraSharp IPS color calibrated screen (3,840 × 2,160 px, 99% sRGB color space coverage) turned into a vertical position to accommodate the life-sized images used. The data were collected via Qualtrics survey suite (Qualtrics, Provo, UT, United States).

Raters were asked to rate fighting ability (“Jak moc by byl tento muž úspěšný, kdyby se dostal do fyzického souboje?”/“If this man were involved in a physical confrontation, how successful would he be?”) of each photograph on a 7-point verbally anchored scale (from “1—velice neúspěšný”/“very unsuccessful,” to “7—velice úspěšný”/“very successful”). The 360° rotations spun automatically once and then raters could freely turn the photographs around for further inspection by dragging the mouse left or right before rating them. Photographs were presented in a randomized order and time spent rating was not restricted. Finally, all raters completed a brief questionnaire (regarding their age, height, weight, and self-rated formidability).

### Physical Performance and Body Composition Measurements

**Figure 2:**
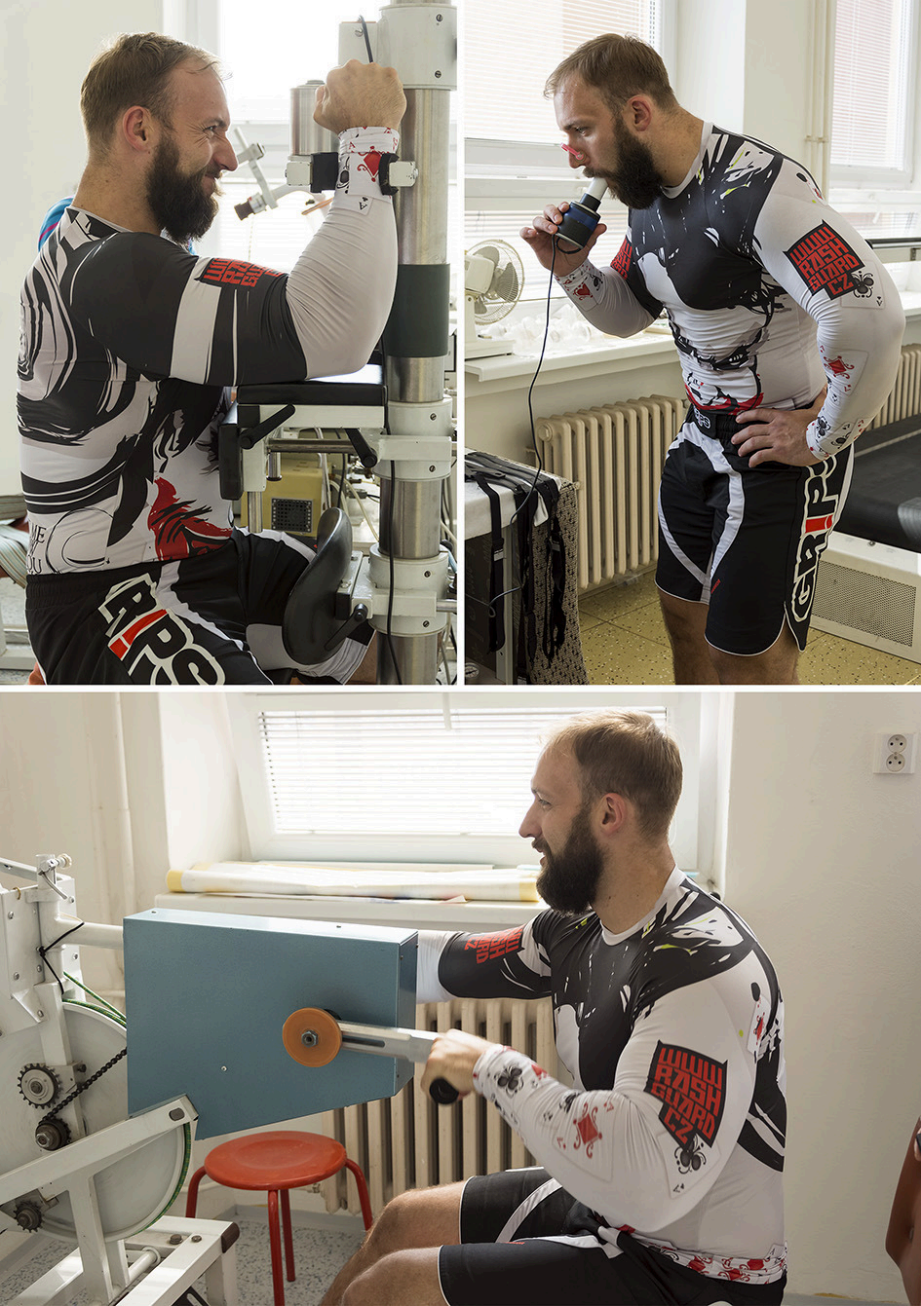
Physical performance measurements. **Top left**—maximal isometric strength (arm extension dynamometry) measurement, **top right**—lung capacity (spirometry) measurement, **bottom**—anaerobic capacity measurement (Wingate test). Photographs by Jitka Fialová, with informed and written consent of the depicted participant.

To determine the physical performance and body parameters of participating athletes, we employed complex measurements relevant to martial arts performance, which included quantifications of their body composition, maximal isometric strength, lung capacity, and AC measurements (Schick et al., 2010; Vidal Andreato et al., 2011; Lenetsky and Harris, 2012; Alm and Yu, 2013; Coufalová et al., 2014; Marinho et al., 2016). Table 1 provides descriptive statistics. All measurements were performed at the Biomedicine Laboratory of the Faculty of Physical Education and Sport, Charles University (see Figure 2).

**Table 1:** Descriptive statistics of the target sample.

**Descriptive statistics**				
**Characteristic**	**Mean**	***SD***	**Minimum**	**Maximum**
Age (yrs)	26.73	5.91	18	38
Height (cm)	179.82	6.93	165	193.8
Body Weight (kg)	81.47	11.3	60.6	112.4
Body Fat (kg)	7.56	4.53	2.5	21.7
Muscle Mass (kg)	70.27	7.68	55	90
Bone Mass (kg)	3.64	0.37	2.9	4.6
Total Body Water (%)	54.03	6.41	43.2	72.9
Handgrip Strength (right) (kp)	56.27	7.89	39.1	77.4
Handgrip Strength (left) (kp)	54.25	7.43	33.2	77.7
Handgrip Strength (mean) (kp)	55.26	7.33	36.15	77.55
Arm Flexion (right) (kp)	34.5	7.01	22.5	60.9
Arm Flexion (left) (kp)	32.69	7.33	20.5	64.4
Arm Flexion (mean) (kp)	33.59	7.05	22.4	62.65
Arm Extension (right) (kp)	28.41	6.12	17.3	54.3
Arm Extension (left) (kp)	28.69	6.26	10.2	51.3
Arm Extension (mean) (kp)	28.55	6.01	13.75	52.8
Trunk Bend (kp)	94.2	18.36	53.7	166.6
Trunk Forward (kp)	80.46	16.6	49.1	141.8
Neck Forward (kp)	24.03	5.12	14.1	33.7
Neck Bend (kp)	40.26	6.01	31.3	57
Knee Flexion (right) (kp)	31.7	8.27	19.8	57.4
Knee Flexion (left) (kp)	30.17	7.4	19.1	53.4
Knee Flexion (mean) (kp)	30.93	7.7	19.6	55.35
Knee Extension (right) (kp)	69.89	16.91	39.7	116.9
Knee Extension (left) (kp)	64.99	13.26	39.6	95.5
Knee Extension (mean) (kp)	67.44	14.56	39.65	105.35
Forced Vital Capacity (l)	5.28	0.73	4.03	6.84
Forced Expiratory Volume (l)	4.59	0.57	3.13	5.66
Peak Expiratory Flow (l/s)	9.55	1.6	6.66	14.28
Maximum Anaerobic Performance (W)	653.28	130.99	422	966
Minimum Anaerobic Performance (W)	364.76	57.94	197.9	514.1
Average Anaerobic Performance (W)	506.7	87.86	293.1	712
Anaerobic Capacity (kJ)	15.2	2.64	8.8	21.4
Decrease of Performance (W)	290.63	100.83	102.2	521.6
Number of Rotations	46.49	5.77	29	57.1
Actual Fighting Ability (wins/fights ratio)	0.65	0.25	0	1
Mean Fighting Ability Rating	4.05	1.01	2.22	6

#### Body Composition Measurements

To acquire detailed measures of body composition, we performed a bioelectrical impedance analysis, which is based on measuring body's electrical resistance to an imperceptible electric current. Electrical resistance is a function of both body shape and the volume of conductive tissues in the body (Goran, 1998). Participating athletes were asked to avoid activities which may bias the measurement, such as consumption of alcoholic beverages, sauna, and demanding physical activities 24 h prior to the test, and eating and drinking for 2 h before the measurement (Brodie et al., 1991; Fogelholm et al., 1993). Body weight, body fat mass, muscle mass, and bone mass were measured (Vaara et al., 2012) using Tanita MC-980 bio impedance scale (Athlete setting). Testing was performed in a standing position, with participants both standing on and holding in their hands measuring electrodes with arms freely alongside the body. Participants were tested while wearing only the underwear we provided them with (Pinilla et al., 1992).

#### Maximal Isometric Strength Measurements

Isometric strength in flexion and extension of arms, legs, trunk, and neck was measured as the peak force produced by maximal voluntary isometric contraction of each muscle group while the athlete was seated on a specifically designed dynamometric station with low profile aluminum load cell (model 1042, measurement error ± 0.05%) (Coufalová et al., 2014). Using a digital hand-grip dynamometer Takei TKK 5401, we evaluated the isometric strength of hands (Vidal Andreato et al., 2011; Bonitch-Góngora et al., 2013). While performing the hand grip measurements, athletes were instructed to stand straight with their arms alongside their body.

Three attempts were performed for each type of measurement while switching sides between attempts and using the “best test” method, meaning that only the highest performance was recorded and included in subsequent analyses.

#### Lung Capacity Measurements

To assess the lung capacity, we used spirometry. This physiological test measures how individuals inhale and exhale volumes of air as a function of time while measuring either total volume or flow. Measures of lung capacity were acquired with spirometer MicroLab ML3500 MK8. Three standing forced vital capacity (FVC) maneuvers were performed: we measured the highest volume of FVC, forced expiratory volume in the first second (FEV1), and peak expiratory flow (PEF), while again applying the “best test” method, i.e., recording the highest of three test values. FVC is the maximal volume of air exhaled with maximally forced effort from a maximal inspiration delivered during an expiration made as forcefully and completely as possible (i.e., vital capacity performed with maximally forced expiratory effort). FEV1 is the maximal volume of air exhaled in the first second of forced expiration from a position of full inspiration and PEF indicates the maximum expiratory flow achieved from maximum forced expiration from the point of maximal lung inflation (Miller et al., 2005).

#### Anaerobic Capacity Measurements

Anaerobic performance we measured using the Wingate test, which consists of 30 s of supramaximal arm-cranking exercise at maximal speed against a frictional resistance determined relative to the subject's body weight (Bar-Or, 1987). Three indices are measured: (1) anaerobic power (AP), which indicates the highest mechanical power elicited during the test, (2) mean power, which shows the average power sustained throughout the 30 s period, (3) AC, which indicates the total work performed during the entire 30 s period, and (4) power decrease (PD), which measures the degree of power drop-off during the test (Collomp et al., 1991). The Wingate test was performed on a Monark arm ergometer (model Rump-Rokos 4.00/C01) with a load of 4 W per kilogram of body weight. Participants were instructed to remain seated and verbally encouraged to perform as quickly as possible right from the start and to maintain maximal turning rates throughout the 30 s period. The test was preceded by a short warm up period, where the participant exercised until achieving 120 bpm heart rate. This was followed by activation of the load (Franchini et al., 2003).

### Level of Beardedness

Two authors coded each target's image. To assess the level of facial hair, we employed three beardedness categories defined in earlier research (Dixson et al., 2018). Agreement between both authors was above 95%, i.e., in 42 out of 44 cases; the remaining two cases were discussed and categorized. The procedure resulted in categories: (1) “Shaved” including athletes with no facial hair of any kind (*N* = 15; 34%); (2) “Some beard” including athletes with all kinds of facial hair except shaven and full beards (*N* = 20; 45.5%); (3) “Full beard” including athletes with trimmed and bushy full beards (*N* = 9; 20.5%).

### Statistical Analysis

All statistical tests were performed in SPSS 23 (IBM Corp., 2015) and JASP 0.9.0.1 (JASP Team, 2018). McDonald's ω statistics was used to estimate inter-rater agreement. Differences in fighting ability ratings were analyzed by independent samples *t*-test and association between ratings was assessed by bivariate correlations using Pearson's correlation coefficient. Component scores of physical performance measures, which were then used in subsequent analyses, were calculated using principal component analysis (PCA) with no rotations. To assess which factors predict the perceived fighting ability and to estimate their relative contribution, we ran a linear regression analysis where all predictors were entered simultaneously using the enter method. Similarly, we used regression analysis to investigate the relationship between the actual and perceived fighting ability. To test the influence of beardedness on fighting ability ratings, one-way ANOVA was carried out. We entered fighting ability ratings as dependent and level of beardedness as independent variables. The effect size for one-way ANOVA is reported in $\eta_{\text{p}}^{2}$. A Holm's *post-hoc* test was performed and effect sizes for the comparison are reported in Cohen's *d*.

#### Component Scores of Physical Performance Measures

In order to reduce the number of variables produced by physical measurements and body composition tests and to obtain robust and representative component scores to apply in subsequent analyses, we used a PCA. We checked the assumptions of this analysis by looking for multicollinearity (>0.9) or singularity (= 0.0) between variables by a bivariate correlation. For body composition measures, we found a high correlation between body weight, body fat, muscle mass, bone mass, and total body water (*r*s > 0.817). For later regression analysis, we have therefore decided to keep body weight as the most representative variable that includes all body composition measures. It is a frequently used measure of body size, thus allowing for a comparison with previous research. Analysis of AC data yielded by the Wingate test measurements revealed a high correlation between maximum performance, average performance, AC, and decrease of performance (*r*s > 0.9). In view of these results, and because we use maximal performance also in other measurements, we decided to use maximum performance as a variable in the PCA. After these initial adjustments, assumptions of the analysis were met.

We subjected maximal isometric strength measurements to the PCA. This produced a single component which we labeled “Isometric strength.” Next, we entered spirometry test measurements into the PCA, which resulted in a component we labeled “Lung capacity.” Anaerobic capacity measurements also yielded a single component, the “Anaerobic capacity.” The resulting components and their loadings are listed in the Supplementary Materials, Table S1.

### Data Availability

Datasets generated and analyzed during the current study are available in the Supplementary Material of this article (Dataset athletes.XLSX, Dataset rating.XLSX).

## Results

McDonald's ω scores of ratings by males (ω = 0.851), females (ω = 0.738), and total (ω = 0.795) showed a high inter-rater agreement. In subsequent analyses, we have therefore used mean fighting ability ratings. We have also found a high correlation between fighting ability ratings assigned by men and women (*r* = 0.972, 95% CI [0.95, 0.985], *p* < 0.001), which is why we decided to analyze the ratings of both sexes together. Independent samples *t*-test also showed no significant sex differences in ratings [*t*_(86)_ = 0.041, *p* = 0.968, *d* = 0.009], which further supported our decision to analyze the ratings of both sexes together.

### Predictors of Perceived Fighting Ability

A multiple linear regression analysis was run to predict perceived fighting ability whereby age, weight, Isometric strength, Lung capacity, and AC components were all treated as independent predictors. The overall model was significant [*F*_(5, 38)_ = 2.79, *p* = 0.031, *R*^2^ = 0.269], but none of the individual predictors statistically significantly predicted the perception of fighting ability: all ps > 0.05 (see Table 2).

**Table 2:** A summary of regression analysis for variables predicting the perceived fighting ability (fighters' age, body weight, Isometric strength, Lung capacity, and Anaerobic capacity component).

**Variable**	**Unstandardized coefficients**		**Standardized coefficients**	***t***	***p***	**95 % CI for B**		**Correlations**		
	**B**	**SE**	**Beta**			**Lower**	**Upper**	**Zero-order**	**Partial**	**Part**
(Constant)	0.931	1.542		0.604	0.549	−2.190	4.052			
Age	0.032	0.029	0.186	1.097	0.279	−0.027	0.090	0.347	0.175	0.152
Body weight	0.028	0.018	0.313	1.572	0.124	−0.008	0.064	0.345	0.247	0.218
Isometric strength component	−0.284	0.181	−0.282	−1.569	0.125	−0.651	0.083	0.095	−0.247	−0.218
Lung capacity component	0.003	0.162	0.003	0.018	0.985	−0.324	0.330	0.098	0.003	0.003
Anaerobic capacity component	0.296	0.182	0.294	1.631	0.111	−0.071	0.664	0.418	0.256	0.226

### Actual Fighting Ability as a Predictor of Perceived Fighting Ability

Exploratory correlation analysis showed a positive correlation between fighter's age (*r* = 0.35, *p* = 0.018), weight (*r* = 0.341, *p* = 0.022), and perceived fighting ability, which is why we added these measures into the linear regression model. The overall model significantly predicted perceived fighting ability [*F*_(3, 40)_ = 3.579, *p* = 0.022, *R*^2^ = 0.212]. Among the predictors, body weight significantly predicted perceived fighting ability (β = 0.31, *t* = 2.033, *p* = 0.049), but actual fighting ability (β = −0.175, *t* = −1.205, *p* = 0.235) nor age (β = 0.247, *t* = 1.669, *p* = 0.103) were statistically significantly related to perceived fighting ability (see Table 3).

**Table 3:** Summary of regression analysis for the relationship between perceived and actual fighting ability, fighters' age, and body weight.

**Variable**	**Unstandardized coefficients**		**Standardized coefficients**	***t***	***p***	**95% CI for B**		**Correlations**		
	**B**	**SE**	**Beta**			**Lower**	**Upper**	**Zero-order**	**Partial**	**Part**
(Constant)	1.141	1.099		1.038	0.306	−1.081	3.363			
Actual fighting ability (wins/fights ratio)	−0.715	0.594	−0.175	−1.205	0.235	−1.915	0.485	−0.109	−0.187	−0.169
Age	0.042	0.025	0.247	1.669	0.103	−0.009	0.093	0.347	0.255	0.234
Body weight	0.028	0.014	0.310	2.033	0.049	0.000	0.055	0.345	0.306	0.285

### The Effect of Beardedness on Perceived Fighting Ability

**Figure 3:**
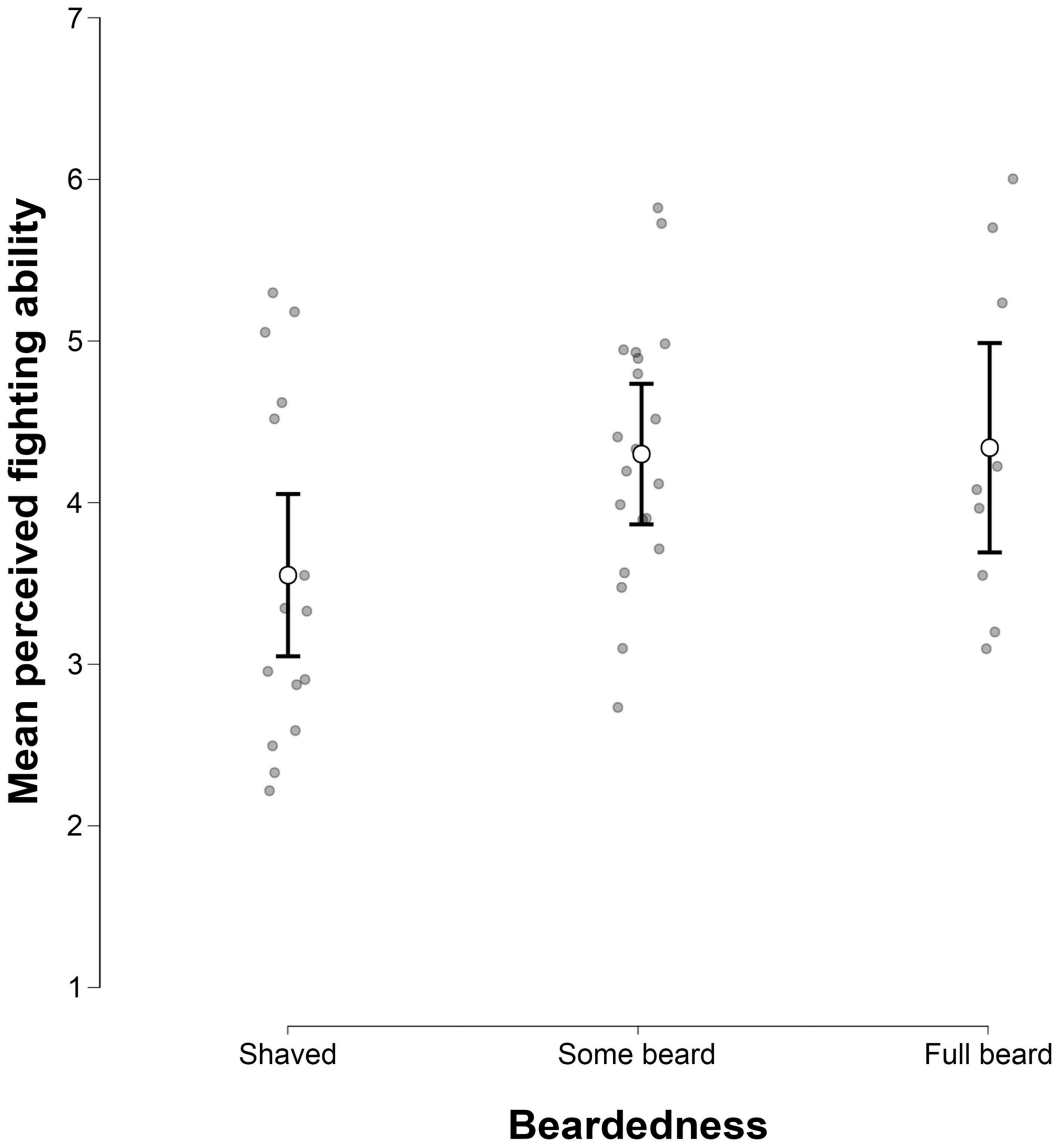
Differences in mean ratings of fighting ability between levels of beardedness (Shaved, Some beard, Full beard). The graph represents the means, their 95% CIs, and data distribution for the three beardedness levels. Mean perceived fighting ability did not differ significantly between beardedness levels.

We found a moderate effect bordering on a formal level of significance of beardedness (Shaved: *M* = 3.55, *SD* = 1.09; Some beard: *M* = 4.3, *SD* = 0.8; Full beard: *M* = 4.34, *SD* = 1.07) on fighting ability rating [*F*_(2, 41)_ = 3.099, *p* = 0.056, $\eta_{\text{p}}^{2}$ = 0.131]. For exploratory purposes, we ran Holm's *post-hoc* comparison. Although not significantly, the Shaved category received the lowest rating, while Some beard (*t* = 2.279, *p*_*Holm*_ = 0.084, Cohen's *d* = 0.801) and Full beard (*t* = 1.943, *p*_*Holm*_ = 0.118, Cohen's *d* = 0.729) categories received higher ratings. Some beard and Full beard categories did not differ (*t* = 0.102, *p*_*Holm*_ = 0.919, Cohen's *d* = 0.044) (Figure 3).

## Discussion

In the present study, we tested which aspects of physical performance affect the perception of fighting ability. To that purpose, we used 360° head rotation photographs of male MMA athletes. We gathered detailed physical measures relevant to physical confrontations, and although it turned out that overall physical performance predicts fighting ability rating, statistical analysis did not show that any particular predictor contributes to the perception of fighting ability significantly. Body weight and AC (AC component) did, however, explained most of the variability, while isometric strength (Isometric strength component) was related negatively. We did not find any significant association between the perceived fighting ability and actual fighting ability in physical confrontations, and perceived fighting ability was predicted solely by athletes' body weight. Further, we explored a possible effect of beardedness on perceived fighting ability and found a moderate-sized but non-significant effect, whereby the shaved targets received the lowest rating.

Compared to previous investigations which used either just one or a limited number of threat potential measures, we collected detailed data about various aspects of physical performance. Although other studies (e.g., Sell et al., 2009; Han et al., 2017) have reported that handgrip strength, height, and weight affect the perception of overall fighting ability, our aim was to investigate other relevant factors which could potentially contribute to perceptual inferences, such as overall isometric strength, anaerobic, or lung capacity. Our regression model significantly predicted perceived fighting ability but none of the individual predictors contributed to the perceived fighting ability significantly. We identified body weight and AC as variables that have the greatest impact on perceived fighting ability. The general probability of being perceived as a winner seems to be related to body size and weight (Deaner et al., 2012), whereby heavier athletes are seen as better fighters than the lighter ones.

It has been suggested in earlier studies that body size (here assessed as body weight) plays a key role during the initial phase of formidability assessments (Třebický et al., 2015). We could thus speculate that our findings are compatible with a model according to which assessment of a potential opponent takes place on multiple levels (Třebický and Havlíček, 2017). The first step, the “fight or flight” decision, seems to depend mainly on opponent's overall size. If, however, the rivals are of a comparable size, another level of assessment may be deployed that could be linked to the perception of other potentially significant characteristics.

Another predictor of perceived fighting ability was AC as measured by the Wingate test. Anaerobic capability has been reported as a key characteristic of successful martial arts athletes (James et al., 2016). MMA is physiologically complex and during contests, fighters deploy a wide range of mechanical and metabolic qualities. Intense striking exchanges are common, but twice as many fights end during highly physically demanding ground fight sequences, that is, when fighters use their wrestling and grappling techniques (Del Vecchio et al., 2011). High-intensity and relatively long engagements are therefore a significant part of the overall performance, and they can be approximated by the Wingate test. Moreover, this measure seems to be related to general physical fitness and performance, which is apparent from its correlation with body composition, isometric strength and lung capacity (see exploratory correlations Table S2 in Supplementary Materials). Earlier research has revealed that cues to strength are present in human faces (Hugill et al., 2009) and individuals' strength is connected to masculinity and dominance ratings (Fink et al., 2007; Windhager et al., 2011), but we found no evidence of a similar relationship for assessments of fighting ability. In fact, our data suggest a rather surprising opposite pattern. Although physical performance is undoubtedly the cornerstone of successful performance, psychological characteristics such as personality, ability to cope with stress, or successful execution of techniques and other skills may also significantly affect fighting ability and success (Gould et al., 1981; Filaire et al., 2001; Radochonski et al., 2011; Ruiz and Hanin, 2011; Chen and Cheesman, 2013; Bernacka et al., 2016). These factors, however, exceed the scope of the current study and should be investigated in future.

Our results are also in contrast with former studies which showed that people can accurately assess actual fighting ability from facial photographs of MMA fighters when asked to rate their aggressiveness, fighting ability, or likelihood of wining (Třebický et al., 2013; Little et al., 2015). Třebický et al. (2013) found a link between the perceived and actual fighting ability only in heavyweight fighters. Limited sample size and uneven distribution within weight categories did not allow us to test the effect of weight category directly, but heavier athletes in our sample were perceived as more formidable competitors, which suggests a similar pattern (see Table S2).

Numerous studies have shown that beards enhance ratings of traits related to intrasexual competition, such as men's perceived age, masculinity, social dominance, or aggressiveness. In short, bearded men tend to score higher in these measures than clean-shaved individuals (Neave and Shields, 2008; Dixson and Vasey, 2012; Geniole and McCormick, 2015; Saxton et al., 2016). Our study provides additional support to these findings because athletes with facial hair were rated higher on the perceived fighting ability scale.

The main goal of present study was to investigate the visual perception of threat potential. We have therefore employed a more holistic concept of fighting ability and did not focus solely on the perception of particular characteristics which may contribute to fighting success. In other studies, participants were asked to assess strength, dominance, masculinity, and aggressiveness, which are all relatively simple characteristics. In the current study, participants rated fighting ability, an arguably more abstract or comprehensive quality, which made the assessments more difficult to process. One could speculate that the use of a different rating scale, e.g., one focused on aggressiveness, could yield significant findings, because earlier studies (e.g., Třebický et al., 2013) have reported a close association between this characteristic and perceived fighting ability.

Future studies should address also non-European populations of raters (Třebický et al., 2018). It is possible that other aspects of the male physique may be associated with perceived fighting ability for instance in Asian cultures, where agility, flexibility, and movement complexity may play a more important role. It is also possible that originally African fighting styles were more dynamic than the rather static and force-oriented European styles.

Our sample substantially differs from previous studies in several aspects. Athletes in the present study varied in performance levels, ranging all the way from beginner amateurs to seasoned professionals, while earlier studies used as stimuli photographs of high-profile professional fighters (UFC). Professional fighters have a considerably greater fighting record, which translates into more accurate estimation of their actual fighting ability. In our study, we included fighters who took part in at least two fights, but this low number of fights may result in an inaccurate picture of athlete's true fighting potential, especially in case of fighters who are just starting their careers. Moreover, reliability of the score could be affected by the way in which fighters are paired for matches, which is not a random process. Fighters are paired by organizing committees who take into account their previous experience and fighting record. This may potentially limit the use of the success score as a measure of actual fighting ability. One could think of a more complex measure of fighting ability which would take into consideration the formidability of the opponent (e.g., winning a fight against an experienced fighter would result in a bonus score, i.e., a higher score than winning a fight with a beginner). Data on formidability/experience of the individual fighters were not, however, obtained in our sample and the wins-to-all-fights ratio remains the most objective measure of the fighting ability available at the moment.

The present study is also based on a relatively small sample and one could therefore argue that it had a rather limited chance of detecting expected associations. Nevertheless, our sample size is comparable to earlier studies such as Han et al. (2017) (*N* = 44); Sell et al. (2009) (*N* = 59) or Windhager et al. (2011) (*N* = 26). Despite our best efforts, we found no more volunteers among fighters who would meet our criteria (age 18–40, at least two MMA fights) and were willing to participate, because the popularity of MMA in the Czech Republic is increasing rather slowly.

To limit a potential systematic bias in photographs and to give our raters maximum visual information, we took highly standardized 360° rotating photographs. We have also asked participants to adopt a neutral expression and straight position during the whole photo session, which could help eliminate possible cues to fighting ability inferences based on slight and unintentional facial expressions. This is an important point because several earlier studies used downloaded photographs of professional fighters, which varied in lighting conditions, head tilts etc. Variability in stimuli quality in previous research could be viewed as “noise” which decreased the likelihood of finding a systematic effect. Nonetheless, can we really take it for granted that this assumption is correct? Could it be that a more formidable depiction, e.g., one with a head tilt and a frown, is a cue to better fighters? The effect of image standardization vs. self-expression on accuracy of inferences should be addressed in future studies.

One may also argue that the generalization potential of results based solely on MMA fighter population may be limited due to its specific characteristics, such as overall high level of formidability and rather specific appearance (broken noses, facial scars, etc.). We tried to decrease potential bias by informing the raters about the target selection criteria only upon completion of the study. Interestingly, the mean formidability rating on a 7-point scale was ≈ 4 (ranging from 2.22 to 6) and the data followed a normal distribution. Although the physical performance of the MMA fighters may be considerably higher than that found in the general population in industrialized countries, it may be less impressive when compared to aged-matched individuals from small-scale societies. It is thus possible that a high level of athletic performance mirrors ancestral human conditions better than the commonly used student samples.

In conclusion, we found no significant connections between the measured predictors of physical performance and the perception of fighting ability from facial photographs. Based on observed effect sizes, we can tentatively conclude that inferences of fighting ability are mostly linked to body size (especially weight) and AC, which are both qualities which affect the outcome of physical confrontations. Our results therefore indicate that the perception of fighting ability may be more complex than previously thought.

## Author Contributions

VT, JF, and JH developed the study concept. KK and DS contributed to the study design. Data collection was performed by VT, JF, DS, KC, RK, ZŠ, and RP. VT and JF performed the data analysis and interpretation. VT, JF, and JH drafted the manuscript. DS, KC, KK, RK, ZŠ, and RP provided critical revisions. All authors approved the final version of the manuscript for submission.

### Conflict of Interest Statement

The authors declare that the research was conducted in the absence of any commercial or financial relationships that could be construed as a potential conflict of interest.

## Supplementary Material

Video 1 Sample of 360° head rotation.

Table S1 Factor analysis; Exploratory correlation matrix.

Table S2 Dataset for formidability rating.

Table S3 Dataset for Athletes measurements.
